# Red Blood Cell Transfusion for Incidence of Retinopathy of Prematurity: Prospective Multicenter Cohort Study

**DOI:** 10.2196/60330

**Published:** 2024-09-18

**Authors:** Xiaoling Wang, Rui Rao, Hua Li, Xiaoping Lei, Wenbin Dong

**Affiliations:** 1 Jinan University, Guangzhou, China; 2 Department of Neonatology, Children’s Medical Center, The Affiliated Hospital of Southwest Medical University, Luzhou, China

**Keywords:** red blood cell transfusion, retinopathy of prematurity, VPI, very preterm infants, ROP, visual impairment, blindness, RBC, red blood cell

## Abstract

**Background:**

Retinopathy of prematurity (ROP) is a leading cause of visual impairment and blindness in preterm infants.

**Objective:**

This study sought to investigate the association between red blood cell (RBC) transfusion and ROP in very preterm infants (VPIs) to inform clinical strategies for ROP prevention and treatment.

**Methods:**

We designed a prospective multicenter cohort study that included VPIs and follow-up data from January 2017 to December 2022 at 3 neonatal clinical medicine centers. They were categorized into a transfusion group (infants who received an RBC transfusion within 4 wk) and a nontransfusion group. The relationship between RBC transfusion and ROP incidence was assessed using binary logistic regression, with subgroup analyses based on gestational age, birth weight, sex, and sepsis status. Inverse probability of treatment weighting and propensity score matching were applied to account for all potential confounding factors that could affect ROP development, followed by sensitivity analysis.

**Results:**

The study included 832 VPIs, including 327 in the nontransfusion group and 505 in the transfusion group. The transfusion group had a lower average birth weight and gestational age and a greater incidence of ROP, ≥stage 2 ROP, and severe ROP. Logistic regression analysis revealed that the transfusion group had a significantly greater risk of ROP (adjusted odds ratio [aOR] 1.70, 95% CI 1.14‐2.53, *P*=.009) and ≥stage 2 ROP (aOR 1.68, 95% CI 1.02‐2.78, *P*=.04) but not severe ROP (aOR 1.75, 95% CI 0.61‐5.02, *P*=.30). The trend analysis also revealed an increased risk of ROP with an increasing number of transfusions and a larger volume of blood transfused (*P* for trend<.001). Subgroup analyses confirmed a consistent trend, with the transfusion group at a higher risk for ROP across all subgroups. Inverse probability of treatment weighting and propensity score matching analyses supported the initial findings.

**Conclusions:**

For VPIs, RBC transfusion significantly increases the risk of ROP, and the risk increases with an increasing number of transfusions and volume of blood transfused.

## Introduction

Retinopathy of prematurity (ROP) is a retinal vasoproliferative disease that occurs in premature and low-birth-weight infants and is the most common cause of blindness and low vision in infants [1,2]. Early detection and treatment are essential for preventing irreversible visual impairment and blindness. The main pathophysiological factor in ROP is an imbalance between proangiogenic and antiangiogenic factors released locally in the retina, resulting in abnormal neovascularization [3].

In premature infants, anemia is caused by multiple factors, including insufficient iron reserves from the mother, nutritional deficiency, a short red blood cell (RBC) lifespan, an immature hematopoietic system, and iatrogenic blood loss. RBC transfusion is one of the main treatments for anemia in premature infants. More than half of premature infants with a gestational age of less than 30 weeks and more than 80% of extremely low-birth-weight infants receive at least 1 RBC transfusion during hospitalization [4]. Although RBC transfusion can significantly improve oxygenation status and promote weight gain in anemic premature infants, the adverse reactions caused by RBC transfusion cannot be ignored. Previous studies have reported that RBC transfusion in premature infants is closely related to the development of necrotizing enterocolitis, bronchopulmonary dysplasia, and abnormal neurological outcomes [5]. However, whether RBC transfusion leads to the development of ROP remains controversial. Prospective and retrospective studies have not provided consistent results [6,7]. ROP may regress naturally or progress to stage 3 or higher, with severe cases requiring laser treatment or intravitreal anti–vascular endothelial growth factor (VEGF) injections [8]. Even for infants with ROP who receive timely treatment and those with ROP below the treatment threshold, there is still a risk of permanent visual impairment [9]. Identifying risk factors that can lead to the development and progression of ROP is crucial for its prevention.

Therefore, the purpose of this study was to determine the correlation between RBC transfusion and the occurrence of ROP, providing new insights for the prevention and treatment of ROP.

## Methods

### Study Participants

#### Inclusion and Exclusion Criteria

A multicenter cohort study of very preterm infants (VPIs) who were admitted to 3 neonatal intensive care units in Sichuan was conducted from January 1, 2017, to December 31, 2022. The exclusion criteria for infants were as follows: (1) severe lethal congenital malformations (such as central nervous system malformations, congenital facial malformations, or congenital heart malformations); (2) participation refusal by parents or guardians; (3) incomplete hospitalization and transfusion records; (4) discharge or death before ROP screening; and (5) irregular follow-up after discharge.

#### Definitions and Diagnostic Criteria for Related Diseases

Early-onset sepsis (≤3 d after birth) and late-onset sepsis (>3 d after birth) were classified based on the timing of onset. The clinical diagnosis of sepsis was based on clinical manifestations and positive results for ≥2 nonspecific blood tests, cerebrospinal fluid examination results consistent with bacterial meningitis, or the detection of bacterial DNA in the blood. A confirmed diagnosis required clinical manifestations and positive blood or cerebrospinal fluid (or other sterile body fluid) cultures [10,11].

Premature rupture of membranes refers to the spontaneous rupture of fetal membranes before labor or before the onset of the first stage of labor [12].

Hypertensive disorders of pregnancy are specific and common diseases in pregnant women and include gestational hypertension, preeclampsia (mild and severe), eclampsia, chronic hypertension with preeclampsia, and chronic hypertension with superimposed gestational hypertension [13].

Gestational diabetes mellitus was defined as meeting any one of the following criteria: a random blood glucose level ≥5.1 mmol/L, a postprandial 1-hour blood glucose level ≥10.0 mmol/L, or a postprandial 2-hour blood glucose level ≥8.5 mmol/L [14].

Small for gestational age (SGA) was defined as a birth weight below the 10th percentile of the average birth weight for infants of the same gestational age and sex according to the Chinese birth weight curve for different gestational ages created by Zhu et al [15,16] in 2018.

Apnea of prematurity was defined as a respiratory pause ≥20 seconds or <20 seconds accompanied by a decrease in heart rate or oxygen saturation [17].

#### Transfusion Criteria for Premature Infants

There is no unified transfusion guideline for anemia in premature infants. The timing and volume of transfusions are usually determined by physicians based on the clinical manifestations, laboratory parameters, and transfusion standards set by each department. Clinical manifestations include poor weight gain or slow growth, irregular breathing, and hemodynamic disturbances. The laboratory parameters include the RBC count, hematocrit level, hemoglobin content, and central and peripheral oxygen saturation. The transfusion dose is 15‐20 mL/kg per session, and if repeated transfusions are needed, blood from the same donor should be used whenever possible.

#### ROP Screening, Diagnosis, and Follow-Up

The initial screening for preterm infants was scheduled between 4 and 5 weeks after birth or at a corrected gestational age of 31 to 32 weeks. The examination was conducted by an experienced ophthalmologist using a RetCam III (Natus Medical Inc), an American-made, wide-angle digital pediatric retinal camera, following a systematic order: the posterior pole, macula, temporal, superior, nasal, and inferior regions.

All the examinations were performed by the same ophthalmologist, who diagnosed and staged ROP according to the severity of the condition; ROP was categorized into stages 1 to 5 [18]. Severe ROP was defined as stage 3 to stage 5 ROP or plus disease in zone I or II [19]. Infants with immature retinal development and no ROP undergo retinal re-examinations every 3 to 4 weeks before peripheral vascularization. The follow-up interval for infants with established ROP was determined according to the severity of the condition.

### Methodology

A standardized questionnaire for obtaining basic information for preterm infants was developed, and data from the infants’ hospital records were documented. All the data were entered independently by 2 individuals using EpiData software (version 3.1; The EpiData Association), with discrepancies resolved by a third party. The following data were collected: (1) maternal information: mode of delivery, gestational diabetes status, hypertensive disorders of pregnancy status, chorioamnionitis status, and premature rupture of membrane status (defined as rupture occurring more than 18 h before delivery); (2) infant information: gestational age, birth weight, 5-minute Apgar score, and SGA status; (3) postnatal conditions: duration of nasal continuous positive airway pressure (nCPAP), use of invasive mechanical ventilation, highest oxygen concentration (sustained for more than 2 h), apnea status, early-onset sepsis status, late-onset sepsis status, invasive ventilation status, and duration of invasive ventilation; (4) transfusion-related indicators: transfusion status, age at first transfusion, number of transfusions, and total blood volume transfused within the first 4 weeks of hospitalization; and (5) outcomes: ROP status, ≥stage 2 ROP status, and severe ROP status.

### Statistical Analysis

Statistical software was used for data description and inference. Categorical data are represented by frequencies and percentages, while continuous data are represented by the mean and SD (for normally distributed data) or median and IQR (for nonnormally distributed data). Chi-square tests were used for group comparisons of categorical data, and independent *2-tailed t* tests or Mann‒Whitney *U* tests were used for continuous data. Multivariate binary logistic regression was used to adjust for potential confounders and calculate the adjusted odds ratio (aOR) of RBC transfusion in premature infants with ROP. Trend tests (*P* for trend) were used to assess the association between the number of transfusions and the volume of blood transfused in infants with ROP. Subgroup analysis was performed based on gestational age, birth weight, sex, and sepsis status. Forest plots were generated to analyze the impact of RBC transfusions on the incidence of ROP in each subgroup.

To address potential baseline data bias in real-world studies, inverse probability of treatment weighting (IPTW) and 1:1 propensity score matching (PSM) methods were used to balance baseline differences between groups [20]. Sensitivity analysis was conducted using the calculated sample sizes obtained from these postrandomization methods to validate the stability of the results. All the statistical analyses were performed using open-source R packages and SPSS software (version 26.0; IBM Corp). A *P* value <.05 was considered to indicate statistical significance.

### Ethical Considerations

The Clinical Trial Ethics Committee of the Affiliated Hospital of Southwest Medical University approved the study (approval number Y2024124). The study was in accordance with the ethical standards of the institutional research committee and with the 1964 Helsinki Declaration and its later amendments. Informed consent was obtained from the legal guardians of all participants included in the study.

## Results

### Case Selection Process

**Figure 1.**
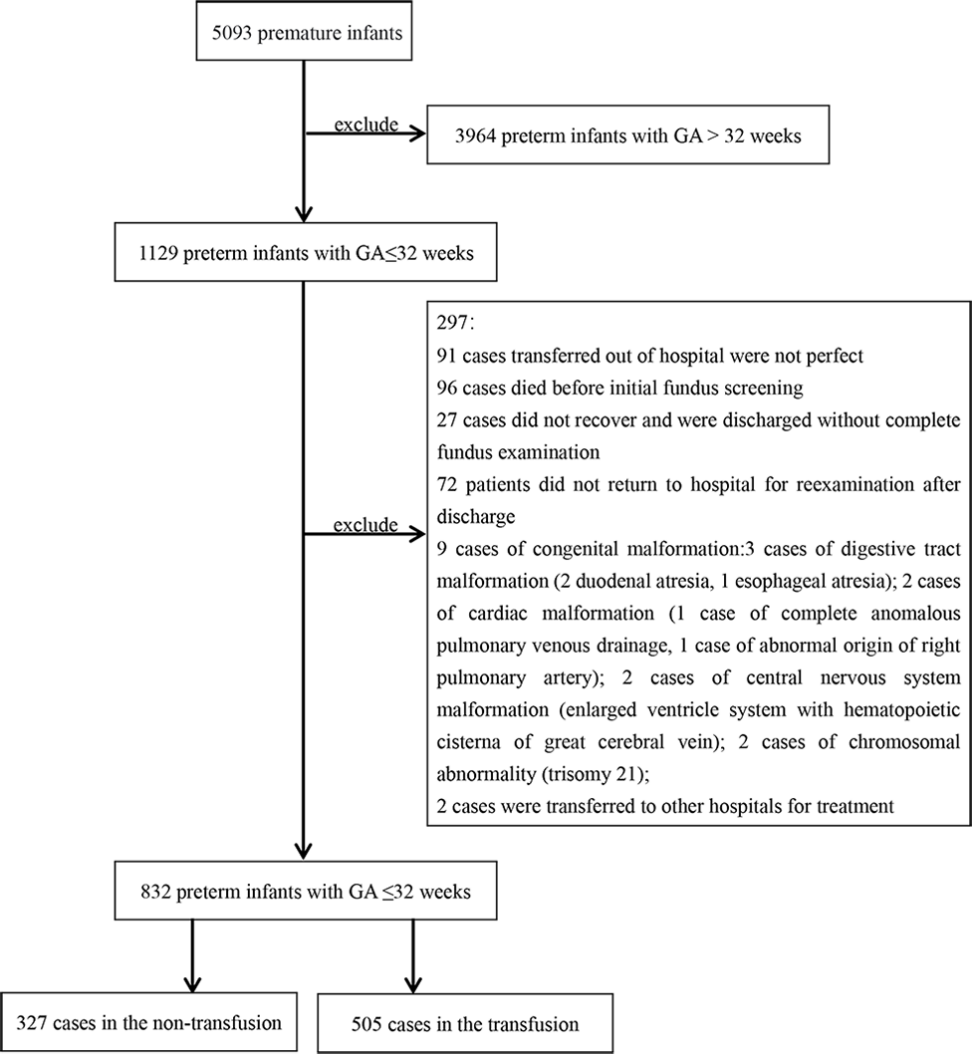
Flowchart of the selection of the research participants. GA: gestational age.

During the study period, a total of 1129 VPIs were admitted to 3 neonatal intensive care units. After excluding 297 infants, 832 were ultimately included. Since the initial screening for ROP occurs between 4 and 5 weeks after birth, infants who received RBC transfusions within 4 weeks after birth were defined as the transfusion group, and those who did not receive transfusions within 4 weeks or who received transfusions after 4 weeks were defined as the nontransfusion group. The nontransfusion group consisted of 327 (39.3%) infants, and the transfusion group included 505 (60.7%) infants (Figure 1).

### Study Results Before Random Matching

#### Comparison of General Information Between the 2 Groups

Table 1 shows that the preterm infants in the transfusion group had a younger gestational age (median 30.10, IQR 28.90-31.10 wk vs median 31.10, IQR 30.30-31.60 wk, z=−8.77, *P*<.001), a lower average birth weight (mean 1327, SD 251 g vs mean 1543, SD 231 g; *t*=12.50, df=830, *P*<.001), and a lower 5-minute Apgar score (median 8, IQR 7-9 points vs median 9, IQR 8-9 points, z=−6.73, *P*<.001). The highest oxygen concentrations required were greater (median 30%, IQR 28-40% vs median 28%, IQR 25-30%, z=−10.02, *P*<.001); the duration of nCPAP use was greater (median 11, IQR 6-18 d vs median 6, IQR 3-9 d, z=−12.03, *P*<.001); and the incidences of mechanical ventilation (156/505, 30.9% vs 16/327, 4.9%, *χ*^2^_1_=81.80, *P*<.001), apnea (280/505, 55.5% vs 87/327, 26.6%, *χ^2^*_1_=66.96, *P*<.001), early-onset sepsis (132/505, 26.1% vs 60/327, 18.4%, *χ^2^*_1_=6.79, *P*=.009), late-onset sepsis (198/505, 39.2% vs 57/327, 17.4%, *χ*^2^_1_=44.28, *P*<.001), and SGA (59/505, 11.7% vs 14/327, 4.3%, *χ*^2^_1_=13.59, *P*<.001) were significantly greater in the transfusion group than in the nontransfusion group (Table 1).

**Table 1.** Comparison of baseline characteristics between the 2 groups.

General information		Nontransfusion group (n=327)	Transfusion group (n=505)	z, chi-square (*df*), or *t* test (*df*)	*P* value
**Maternal data, n (%)**					
	Vaginal delivery	183 (56.0)	264 (52.3)	1.09(1)	.30
	Premature rupture of membrane	143 (43.7)	154 (30.5)	15.15(1)	<.001
	Hypertensive disorders of pregnancy	38 (11.6)	72 (14.3)	1.20(1)	.27
	Gestational diabetes mellitus	68 (20.8)	125 (24.8)	1.75(1)	.19
	Chorioamnionitis	120 (36.7)	177 (35.1)	0.24(1)	.63
**Neonatal data**					
	Male, n (%)	183 (56.0)	282 (55.9)	0.001(1)	.97
	Gestational age (weeks), median (IQR)	31.10 (30.30-31.60)	30.10 (28.90-31.10)	−8.77	<.001
	Birth weight (g), mean (SD)	1543 (231)	1327 (251)	12.50(830)	<.001
	5-min Apgar score, median (IQR)	9 (8-9)	8 (7-9)	−6.73	<.001
	nCPAP^a^ duration (days), median (IQR)	6 (3-9)	11 (6-18)	−12.03	<.001
	Invasive ventilation, n (%)	16 (4.9)	156 (30.9)	81.80 (1)	<.001
	Invasive ventilation duration (days), median (IQR)	0 (0-0)	0 (0-2)	−9.21	<.001
	Maximum oxygen concentration (%), median (IQR)	28 (25-30)	30 (28-40)	−10.02	<.001
	Apnea, n (%)	87 (26.6)	280 (55.5)	66.96 (1)	<.001
	Early-onset sepsis, n (%)	60 (18.4)	132 (26.1)	6.79 (1)	.009
	Late-onset sepsis, n (%)	57 (17.4)	198 (39.2)	44.28 (1)	<.001
	SGA^b^, n (%)	14 (4.3)	59 (11.7)	13.59 (1)	<.001

a nCPAP: nasal continuous positive airway pressure.

b SGA: small for gestational age.

#### The Impact of Transfusion Within 4 Weeks on ROP Incidence

Using the results of univariate analysis as a basis, potential confounding factors were included in a multivariate binary logistic regression model. The results showed that the risk of ROP (aOR 1.70, 95% CI 1.14‐2.53; *P=*.009) and ≥stage 2 ROP (aOR 1.68, 95% CI 1.02‐2.78; *P=*.04) in the transfusion group was significantly greater than that in the nontransfusion group; however, there was no significant difference in the risk of severe ROP between the 2 groups (aOR 1.75, 95% CI 0.61‐5.02; *P=*.30) (Table 2).

**Table 2.** The impact of red blood cell (RBC) transfusion within 4 wk on retinopathy of prematurity (ROP) incidence.

	Nontransfusion group (n=327), n (%)	Transfusion group (n=505), n (%)	OR^a^ (95% CI)	*P* value	aOR^b^ (95% CI)	*P* value
ROP	58 (17.7)	239 (47.3)	4.17 (2.99-5.81)	<.001	1.70 (1.14-2.53)	.009
≥Stage 2 ROP	30 (9.2)	159 (31.5)	4.55 (2.99-6.92)	<.001	1.68 (1.02-2.78)	.04
Severe ROP	5 (1.5)	51 (10.1)	7.23 (2.86-18.33)	<.001	1.75 (0.61-5.02)	.30

a OR: odds ratio.

b aOR: adjusted odds ratio. Adjusted for gestational age, birth weight, 5-min Apgar score, mechanical ventilation use, maximum oxygen concentration, early-onset sepsis, late-onset sepsis, apnea, and small for gestational age.

#### The Impact of Different Transfusion Frequencies Within 4 Weeks on ROP Incidence

To clarify the impact of different transfusion frequencies within 4 weeks on ROP incidence, preterm infants in the transfusion group were further divided into a single transfusion group (339 infants) and a multiple transfusion group (166 infants) and compared with those in the nontransfusion group. A single transfusion within 4 weeks increased the risk of ROP (aOR 1.79, 95% CI 1.20‐2.68; *P*=.005) but did not increase the risk of ≥stage 2 ROP or severe ROP; multiple transfusions within 4 weeks increased the risk of ROP (aOR 2.95, 95% CI 1.67‐5.21; *P*<.001) and ≥stage 2 ROP (aOR 2.84, 95% CI 1.47‐5.47; *P*=.002) but did not increase the risk of severe ROP (Multimedia Appendix 1). As the number of transfusions increased, the risk of ROP, ≥stage 2 ROP, and severe ROP gradually increased, with trend test results all less than 0.001, indicating that the higher the number of transfusions was, the greater the risk of ROP.

#### The Impact of Different Transfusion Volumes Within 4 Weeks on ROP Incidence

To clarify the impact of different transfusion volumes within 4 weeks on ROP incidence, preterm infants in the transfusion group were further divided based on the total volume of blood transfused. Due to the nonnormal distribution of the total transfusion volume within 4 weeks, the infants in the transfusion group were divided into the following 3 groups according to the IQR and compared with those in the nontransfusion group: the ≤34 mL/kg group (176 infants), 34‐42 mL/kg group (159 infants), and ≥42 mL/kg group (170 infants). A total transfusion volume ≤34 mL/kg within 4 weeks did not increase the risk of ROP, ≥stage 2 ROP, or severe ROP; a total transfusion volume of 34‐42 mL/kg within 4 weeks increased the risk of ROP (aOR 1.67, 95% CI 1.02‐2.72, *P*=.04) but did not increase the risk of ≥stage 2 ROP or severe ROP; and a total transfusion volume ≥42 mL/kg within 4 weeks increased the risk of ROP (odds ratio [OR] 2.88, 95% CI 1.54‐5.39, *P*<.001) and ≥stage 2 ROP (OR 3.08, 95% CI 1.53‐6.23, *P=.002*) but did not increase the risk of severe ROP (Multimedia Appendix 2). As the total transfusion volume increased, the risk of ROP, ≥stage 2 ROP, and severe ROP gradually increased, with trend test results all less than 0.001, indicating that the greater the total transfusion volume was, the greater the risk of ROP.

#### The Impact of Transfusion on ROP Incidence in Various Subgroups of Preterm Infants

**Figure 2.**
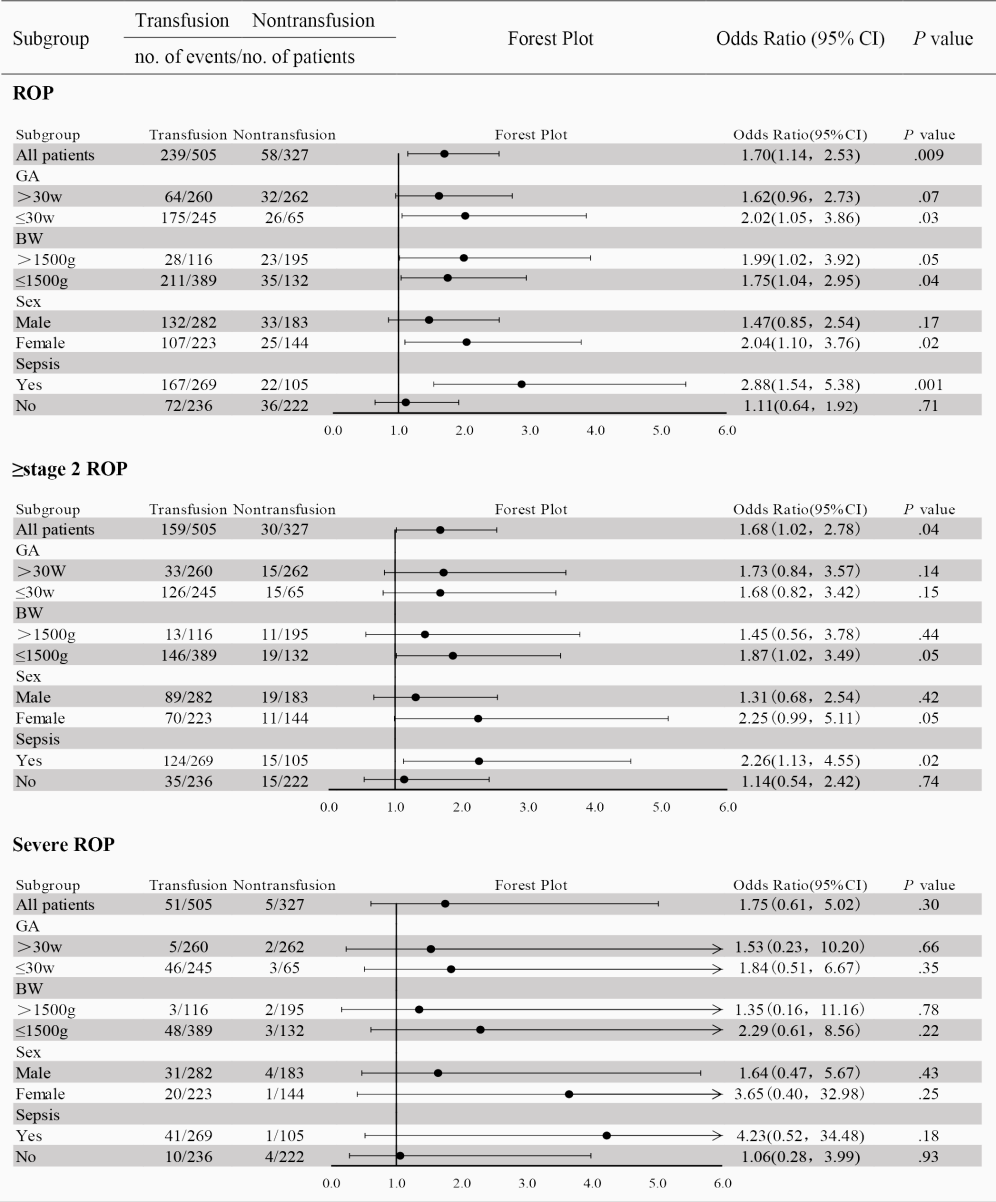
Forest plot of the effect of red blood cell transfusion within 4 wk on the occurrence of retinopathy of prematurity (ROP) in preterm infants in each subgroup. BW: birth weight; GA: gestational age.

To clarify the impact of transfusion within 4 weeks on ROP incidence in different preterm infant populations, the included preterm infants were divided into 3 subgroups based on gestational age (>30 weeks ≤30 weeks), birth weight (>1500 g, ≤1500 g), sex (male, female) and sepsis status (yes, no). Although there were no significant differences in some outcome indicators between the subgroups, the overall trend was consistent, with the risk of ROP in the transfusion group being greater than that in the nontransfusion group (Figure 2, Multimedia Appendices 3, 4, and 5).

### Sensitivity Analysis Results After IPTW and PSM

Given the numerous factors influencing ROP incidence and the significant imbalance in the baseline data in this real-world study, as shown in Table 2, the ROP data showed considerable changes before and after adjusting for confounding factors, suggesting potential selection bias in the study results. Therefore, the IPTW and PSM methods were applied in this study to evaluate all factors that could affect ROP incidence between the 2 groups. Sensitivity analysis was performed using the calculated sample sizes obtained after IPTW and PSM to validate the stability of the impact of RBC transfusions on ROP incidence. The balance between the groups was significantly improved after IPTW and PSM (Multimedia Appendices 6 and 7). The results obtained after IPTW and PSM were consistent with the original results (Multimedia Appendices 8 and 9).

## Discussion

### Principal Findings

Previous studies on the association between RBC transfusion and ROP incidence have used a retrospective case-control design to investigate risk factors for ROP development. This is the first prospective multicenter cohort study in which infants were categorized based on whether they received RBC transfusions within the first 4 weeks after birth. The study included all VPIs who were admitted to 3 centers over nearly 6 years, and the correlation between RBC transfusion and ROP incidence was explored. The results indicated that RBC transfusion within the first 4 weeks significantly increases the risk of ROP in VPIs, and this correlation remained consistent across different groups of preterm infants.

Despite various available measures to prevent anemia in preterm infants, the use of RBC transfusion remains inevitable [21]. Studies show that more than 90% of extremely low-birth-weight infants receive at least 1 RBC transfusion during their hospital stay, with the risk of transfusion increasing for infants with younger gestational ages, lower birth weights, and greater immaturity [22,23]. Puia-Dumitrescu et al’s [24] study, which included 54 preterm infants with a gestational age of 30 weeks and a birth weight less than 1000 g, revealed that the average number of RBC transfusions during a 10-week hospital stay was 8. For extremely preterm infants with a gestational age of 23‐24 weeks, the average number of transfusions was 11, and for those with a gestational age of 27‐28 weeks, the number could reach 6. Our study revealed that 505 (60.7%) preterm infants with a gestational age ≤32 weeks received at least 1 RBC transfusion within 28 days of hospitalization, with a greater likelihood of transfusion in infants with a younger gestational age. The proportion of transfusions decreased with increasing gestational age and birth weight, which is consistent with previous findings.

ROP is a common complication in preterm infants, and its incidence and severity decrease as gestational age and birth weight increase. In this study, the incidence of ROP in preterm infants with a gestational age ≤32 weeks was 297 (35.6%), and the incidence of severe ROP was 56 (6.7%). The incidence of ROP is greater in infants with younger gestational ages, with those born at less than 28 weeks having an incidence rate of approximately 70%, which is consistent with the reports by Dai et al [25].

The pathogenesis of ROP is multifactorial, with low gestational age, low birth weight, and prolonged exposure to oxygen therapy being the main risk factors for ROP [26]. In addition to these risk factors, RBC transfusion has been suggested to be a risk factor for ROP development in numerous case-control studies [27-29]. However, Lundgren et al [30] reported that the duration of anemia in the first week after birth was an independent risk factor for ROP, while RBC transfusion was not. Given the inconsistent results of retrospective studies, our prospective cohort study aimed to clarify the association between RBC transfusion and ROP incidence. The results indicated that after adjusting for confounding factors such as gestational age at birth, birth weight, and nCPAP use, RBC transfusion within the first 4 weeks of hospitalization significantly increased the risk of ROP and ≥stage 2 ROP in VPIs. However, there was no significant difference in the incidence of severe ROP between the 2 groups, which may have been due to the low incidence of severe ROP and the small sample size. Given that this study was observational, many factors influence ROP development, and there is potential selection bias due to the imbalance of baseline data, we applied the IPTW and PSM methods to account for all potential confounding factors that could affect ROP development. The results still suggested that RBC transfusion increases the risk of ROP in VPIs, and this association remained consistent across different groups of preterm infants.

The risk of ROP increases with an increasing number of RBC transfusions and volume of blood transfused. Ghirardello et al’s [31] study, which included 641 preterm infants with very low birth weight, showed that RBC transfusion is an independent risk factor for ROP development in very low-birth-weight infants, and the risk of complications increases with an increasing number of transfusions, with 3 or more transfusions increasing the risk of bronchopulmonary dysplasia and ROP by 4.88 times. Hengartner et al’s [32] retrospective study of 178 extremely preterm infants showed that infants with ≥stage 2 ROP received more transfusions, had larger volumes transfused, and received earlier treatment. Additionally, Uberos et al’s [19] data indicate that the relationship between the number of RBC transfusions and the risk of ROP and severe ROP is more significant than that between early RBC transfusion (within the first 7 d of life) and the risk of ROP and severe ROP. Our study revealed that the risk of ROP and ≥stage 2 ROP increases with an increasing number of RBC transfusions and volume of blood transfused, which is consistent with the findings of the aforementioned studies.

The mechanism by which RBC transfusion leads to ROP development is not yet fully understood, but it may be related to the replacement of fetal hemoglobin (HbF) with adult hemoglobin after transfusion, which leads to changes in the type and quantity of hemoglobin [33]. Since adult hemoglobin has a decreased affinity for oxygen, developing retinal tissue is exposed to high oxygen levels, leading to oxidative damage to vascular endothelial cells, downregulation of VEGF expression, and stagnation of retinal vascular development [34]. This results in retinal hypoxia, increased VEGF and erythropoietin expression, pathological proliferation of vessels in the retina and vitreous, and the development of ROP [35]. Jiramongkolchai et al’s [36] prospective cohort study showed that infants with the lowest percentage of HbF at a corrected age of 31 weeks had a 7.6-fold increased risk of mild and severe ROP, and this risk increased to 12.3 times by the corrected age of 34 weeks, indicating that a lower HbF percentage is associated with a greater risk of ROP. Prasad et al [37,38] conducted 2 prospective studies to explore the correlation between HbF concentration and ROP, and the results showed that preterm infants with lower HbF levels were at higher risk of developing ROP, and preterm infants with higher HbF concentration were more likely to have ROP spontaneously subside. HbF may play a protective role in the occurrence and development of ROP. Teofili et al investigated the relationship between transfusion-free survival and ROP [39]. The study found that preterm infants receiving RBC transfusions before 28 weeks of gestational age were associated with an increased risk of developing severe ROP, that gestational age at second transfusion was a better predictor of developing severe ROP than gestational age at first transfusion, and that maintaining higher levels of HbF may help reduce the risk of ROP [39].

Preventing anemia and reducing the risk of RBC transfusion are pressing issues for neonatologists. Delayed cord clamping [40], reduced iatrogenic blood loss, and iron supplementation are beneficial interventions for preventing anemia. Research on the RBC source, storage time, and different transfusion thresholds is currently ongoing, but there are no unified results. Kirpalani et al [41] reported that restrictive transfusion strategies did not reduce the incidence of ROP; Glaser et al’s [42] large cohort study revealed that restrictive transfusion strategies could reduce the risk of ROP, while liberal transfusion strategies increased the risk. In recent years, umbilical cord blood (UCB) transfusion has gained increasing attention. The use of UCB from healthy newborns has been proposed to prevent ROP in preterm infants. The main potential advantage of UCB is that it contains the same amount of HbF as newborns in utero. Thus, autologous UCB transfusions would maintain a physiological concentration of HbF during the first weeks of life, which has a greater affinity for oxygen and is more stable in an oxidative environment, maintaining similar oxygen transportation and tissue delivery as in the fetal stage. This fact could optimize the postnatal development of different immature tissues [43].

### Limitations

This study has certain limitations. First, as a multicenter observational study spanning 6 years, there may have been changes in the treatment and transfusion strategies for preterm infants across centers, leading to potential bias in the results. Second, although the IPTW and PSM methods were used to control for known confounding factors, unknown confounding factors and other intermediate factors may still have affected the results. Therefore, considering the potential impact of unknown confounding factors, we believe that well-designed, larger-sample randomized clinical trials are needed to further validate the effects of RBC transfusion, different RBC storage times, transfusion intervals, and anemia status on the development of ROP. We also need to learn from the work of Schallmoser et al [44], integrating the medical profession with machine learning to construct a predictive model for the risk of retinopathy of ROP occurrence.

### Conclusions

Based on our study results, RBC transfusion increases the risk of ROP, and this effect is consistent across different groups of preterm infants. For VPIs, RBC transfusions are strongly associated with an increased risk of ROP, which escalates alongside the number and volume of transfusions. We recommend that for VPIs with small gestational age, low birth weight, and high risk of sepsis, the indications for RBC transfusion in preterm infants should be strictly controlled and that the number of transfusions and volume of blood transfused should be limited to reduce adverse consequences.

## Supplementary material

10.2196/60330 Multimedia Appendix 1 The impact of different transfusion frequencies within 4 weeks on retinopathy of prematurity incidence.

10.2196/60330 Multimedia Appendix 2 The impact of different transfusion volumes within 4 weeks on retinopathy of prematurity incidence.

10.2196/60330 Multimedia Appendix 3 The impact of red blood cell transfusion within 4 weeks on retinopathy of prematurity incidence for different gestational ages.

10.2196/60330 Multimedia Appendix 4 The impact of red blood cell transfusion within 4 weeks on retinopathy of prematurity incidence for different birth weights.

10.2196/60330 Multimedia Appendix 5 The impact of red blood cell transfusion within 4 weeks on retinopathy of prematurity incidence for each sex.

10.2196/60330 Multimedia Appendix 6 Change in standardized mean difference (SMD) values before and after inverse probability of treatment weighting (IPTW) and propensity score matching (PSM). (A) Change in SMD values before and after IPTW; (B) change in SMD values before and after PSM.

10.2196/60330 Multimedia Appendix 7 Propensity score value distribution between groups before and after probability of treatment weighting (IPTW) and propensity score matching (PSM). (A) Propensity score value distribution between groups before random matching; (B) propensity score value distribution between groups after IPTW; (C) propensity score value distribution between groups after PSM.

10.2196/60330 Multimedia Appendix 8 The impact of red blood cell transfusion within 4 weeks on retinopathy of prematurity incidence after inverse probability of treatment weighting.

10.2196/60330 Multimedia Appendix 9 The impact of red blood cell transfusion within 4 weeks on retinopathy of prematurity incidence after propensity score matching.

## Abbreviations

aOR: adjusted odds ratio
HbF: fetal hemoglobin
IPTW: inverse probability of treatment weighting
nCPAP: nasal continuous positive airway pressure
OR: odds ratio
PSM: propensity score matching
RBC: red blood cell
ROP: retinopathy of prematurity
SGA: small for gestational age
UCB: umbilical cord blood
VEGF: vascular endothelial growth factor
VPI: very preterm infant
