# Supplementary material for: Red Blood Cell Transfusion for Incidence of Retinopathy of Prematurity: Prospective Multicenter Cohort Study
Source: JMIR Pediatr Parent. 2024 Sep 18;7:e60330. doi: 10.2196/60330 (PMC11425406; doi:10.2196/60330)
Supplement: Multimedia Appendix 1 [file pediatrics-v7-e60330-s001.docx]

Supplementary Table S1. The impact of different transfusion frequencies within 4 weeks on ROP incidence.

|  | N=832, n (%) | OR (95% CI) | *P* value | aOR^a^ (95% CI) | *P* value |
| --- | --- | --- | --- | --- | --- |
| **ROP** | | | | | |
| Nontransfusion group | 58 (7.0) | 1 |  | 1 |  |
| 1 RBC transfusion | 126 (15.1) | 2.74(1.92, 3.93) | <.001 | 1.79 (1.20, 2.68) | .005 |
| >1 RBC transfusion | 113 (13.6) | 9.71(6.31, 14.93) | <.001 | 2.95 (1.67, 5.21) | <.001 |
| *P* for trend | <.001 | | | | |
| **≥stage 2 ROP** | | | | | |
| Nontransfusion group | 30 (3.6) | 1 |  | 1 |  |
| 1 RBC transfusion | 73 (8.8) | 2.72(1.72, 4.29) | <.001 | 1.64 (0.96, 2.79) | .069 |
| >1 RBC transfusion | 86 (10.3) | 10.51(6.49, 17.03) | <.001 | 2.84 (1.47, 5.47) | .002 |
| *P* for trend | <.001 | | | | |
| **Severe ROP** | | | | | |
| Nontransfusion group | 5 (0.6) | 1 |  | 1 |  |
| 1 RBC transfusion | 19 (2.3) | 3.82 (1.41, 10.37) | .008 | 1.30 (0.42, 4.01) | .647 |
| >1 RBC transfusion | 32 (3.8) | 15.27 (5.82, 40.02) | <.001 | 3.32 (0.90, 12.22) | .072 |
| *P* for trend | <.001 | | | | |
| ^a^aOR：adjusted odds ratio. Adjusted for gestational age, birth weight, 5-minute Apgar score, mechanical ventilation use, maximum oxygen concentration, early-onset sepsis, late-onset sepsis, apnea, and SGA. | | | | | |
