# Supplementary material for: Red Blood Cell Transfusion for Incidence of Retinopathy of Prematurity: Prospective Multicenter Cohort Study
Source: JMIR Pediatr Parent. 2024 Sep 18;7:e60330. doi: 10.2196/60330 (PMC11425406; doi:10.2196/60330)
Supplement: Multimedia Appendix 2 [file pediatrics-v7-e60330-s002.docx]

Supplementary Table S2. The impact of different transfusion volumes within 4 weeks on ROP incidence.

|  | N=832, n (%) | OR (95% CI) | *P* value | aOR^a^ (95% CI) | *P* value |
| --- | --- | --- | --- | --- | --- |
| **ROP** | | | | | |
| Nontransfusion group | 58 (7.0) | 1 |  | 1 |  |
| ≤ 34 ml/kg group | 58 (7.0) | 2.28 (1.49, 3.48) | <.001 | 1.52 (0.92, 2.50) | .101 |
| 34-42 ml/kg group | 60 (7.2) | 2.76 (1.81, 4.20) | <.001 | 1.67 (1.02, 2.72) | .041 |
| ≥42 ml/kg group | 121 (14.5) | 11.45 (7.40, 17.72) | <.001 | 2.88 (1.54, 5.39) | .001 |
| *P* for trend | <.001 | | | | |
| **≥stage 2 ROP** | | | | | |
| Nontransfusion group | 30 (3.6) | 1 |  | 1 |  |
| ≤ 34 ml/kg group | 30 (3.6) | 2.31 (1.35, 3.96) | .002 | 1.25 (0.66, 2.36) | .489 |
| 34-42 ml/kg group | 35 (4.2) | 2.46 (1.45, 4.16) | .001 | 1.65 (0.87, 3.10) | .124 |
| ≥42 ml/kg group | 94 (11.3) | 12.25 (7.56, 19.83) | <.001 | 3.08 (1.53, 6.23) | .002 |
| *P* for trend | <.001 | | | | |
| **Severe ROP** | | | | | |
| Nontransfusion group | 5 (0.6) | 1 |  | 1 |  |
| ≤ 34 ml/kg group | 10 (1.2) | 3.88 (1.31, 11.54) | .015 | 1.36 (0.37, 5.08) | .646 |
| 34-42 ml/kg group | 7 (0.8) | 3.20 (1.03, 9.94) | .044 | 1.62 (0.46, 5.73) | .451 |
| ≥42 ml/kg group | 34 (4.1) | 16.10 (6.17, 42.04) | <.001 | 2.89 (0.82, 10.27) | .100 |
| *P* for trend | <.001 | | | | |
| ^a^aOR: adjusted odds ratio. Adjusted for gestational age, birth weight, 5-minute Apgar score, mechanical ventilation use, maximum oxygen concentration, early-onset sepsis, late-onset sepsis, apnea, and SGA. | | | | | |
