# Supplementary material for: Red Blood Cell Transfusion for Incidence of Retinopathy of Prematurity: Prospective Multicenter Cohort Study
Source: JMIR Pediatr Parent. 2024 Sep 18;7:e60330. doi: 10.2196/60330 (PMC11425406; doi:10.2196/60330)
Supplement: Multimedia Appendix 3 [file pediatrics-v7-e60330-s003.docx]

Supplementary Table S3. The impact of RBC transfusion within 4 weeks on ROP incidence of different gestational ages.

|  | Nontransfusion group | Transfusion group | OR (95% CI) | *P* value | aOR^a^ (95% CI) | *P* value |
| --- | --- | --- | --- | --- | --- | --- |
| **GA>30W n=522, n (%)** | | | | | | |
| ROP | 32 (6.1) | 64 (12.3) | 2.35 (1.47, 3.74) | <.001 | 1.62 (0.96, 2.73) | .073 |
| ≥stage 2 ROP | 15 (2.9) | 33 (6.3) | 2.39 (1.27, 4.52) | .007 | 1.73 (0.84, 3.57) | .140 |
| Severe ROP | 2 (0.4) | 5 (1.0) | 2.55 (0.49, 13.26) | .266 | 1.53 (0.23, 10.20) | .663 |
| **GA≤30W n=310, n (%)** | | | | | | |
| ROP | 26 (8.4) | 175 (56.5) | 3.75 (2.12, 6.62) | <.001 | 2.02 (1.05, 3.86) | .034 |
| ≥stage 2 ROP | 15 (4.8) | 126 (40.6) | 3.53 (1.88, 6.62) | <.001 | 1.68 (0.82, 3.42) | .153 |
| Severe ROP | 3 (1.0) | 46 (14.8) | 4.78 (1.44, 15.89) | .011 | 1.84 (0.51, 6.67) | .351 |
| ^a^aOR: adjusted odds ratio. Adjusted for gestational age, birth weight, 5-minute Apgar score, mechanical ventilation use, maximum oxygen concentration, early-onset sepsis, late-onset sepsis, apnea, and SGA. | | | | | | |
