# Supplementary material for: Red Blood Cell Transfusion for Incidence of Retinopathy of Prematurity: Prospective Multicenter Cohort Study
Source: JMIR Pediatr Parent. 2024 Sep 18;7:e60330. doi: 10.2196/60330 (PMC11425406; doi:10.2196/60330)
Supplement: Multimedia Appendix 4 [file pediatrics-v7-e60330-s004.docx]

Supplementary Table S4. The impact of RBC transfusion within 4 weeks on ROP incidence of different birth weights.

|  | Nontransfusion group | Transfusion group | OR (95% CI) | *P* value | aOR^a^ (95% CI) | *P* value |
| --- | --- | --- | --- | --- | --- | --- |
| **GA>1500g (n=311), n (%)** | | | | | | |
| ROP | 23 (7.4) | 28 (9.0) | 2.38 (1.29, 4.37) | .005 | 1.99 (1.02, 3.92) | .045 |
| ≥stage 2 ROP | 11 (3.5) | 13 (4.2) | 2.11 (0.91, 4.88) | .081 | 1.45 (0.56, 3.78) | .444 |
| Severe ROP | 2 (0.6) | 3 (1.0) | 2.56 (0.42, 15.56) | .307 | 1.35(0.16, 11.16) | .780 |
| **GA≤1500g (n=521), n (%)** | | | | | | |
| ROP | 35 (6.7) | 211 (40.5) | 3.29 (2.12, 5.07) | <.001 | 1.75(1.04, 2.95) | .035 |
| ≥stage 2 ROP | 19 (3.6) | 146 (28.0) | 3.57 (2.11, 6.06) | <.001 | 1.87(1.02, 3.49) | .045 |
| Severe ROP | 3 (0.6) | 48 (9.2) | 6.05 (1.85, 19.77) | .003 | 2.29(0.61, 8.56) | .218 |
| ^a^aOR: adjusted odds ratio. Adjusted for gestational age, birth weight, 5-minute Apgar score, mechanical ventilation use, maximum oxygen concentration, early-onset sepsis, late-onset sepsis, apnea, and SGA. | | | | | | |
