# Supplementary material for: Red Blood Cell Transfusion for Incidence of Retinopathy of Prematurity: Prospective Multicenter Cohort Study
Source: JMIR Pediatr Parent. 2024 Sep 18;7:e60330. doi: 10.2196/60330 (PMC11425406; doi:10.2196/60330)
Supplement: Multimedia Appendix 5 [file pediatrics-v7-e60330-s005.docx]

Supplementary Table S5. The impact of RBC transfusion within 4 weeks on ROP incidence of different sex.

|  | Nontransfusion group | Transfusion group | OR (95% CI) | *P* value | aOR^a^ (95% CI) | *P* value |
| --- | --- | --- | --- | --- | --- | --- |
| **Male (n=465), n (%)** | | | | | | |
| ROP | 33 (7.1) | 132 (28.4) | 4.00 (2.57, 6.23) | <.001 | 1.47 (0.85, 2.54) | .171 |
| ≥stage 2 ROP | 19 (4.1) | 89 (19.1) | 3.98 (2.33, 6.81) | <.001 | 1.31 (0.68, 2.54) | .419 |
| Severe ROP | 4 (0.9) | 31 (6.7) | 5.53 (1.92, 15.93) | .002 | 1.64 (0.47, 5.67) | .433 |
| **Female (n=367), n (%)** | | | | | | |
| ROP | 25 (6.8) | 107 (29.2) | 4.39 (2.65, 7.26) | <.001 | 2.04 (1.10, 3.76) | .024 |
| ≥stage 2 ROP | 11 (3.0) | 70 (29.1) | 5.53 (2.81, 10.88) | <.001 | 2.25 (0.99, 5.11) | .053 |
| Severe ROP | 1 (0.3) | 20 (5.4) | 14.09 (1.87, 107.17) | .010 | 3.65 (0.40, 32.98) | .250 |
| ^a^aOR: adjusted odds ratio. Adjusted for gestational age, birth weight, 5-minute Apgar score, mechanical ventilation use, maximum oxygen concentration, early-onset sepsis, late-onset sepsis, apnea, and SGA. | | | | | | |
