# Supplementary figures and images for: Red Blood Cell Transfusion for Incidence of Retinopathy of Prematurity: Prospective Multicenter Cohort Study
Source: JMIR Pediatr Parent. 2024 Sep 18;7:e60330. doi: 10.2196/60330 (PMC11425406; doi:10.2196/60330)

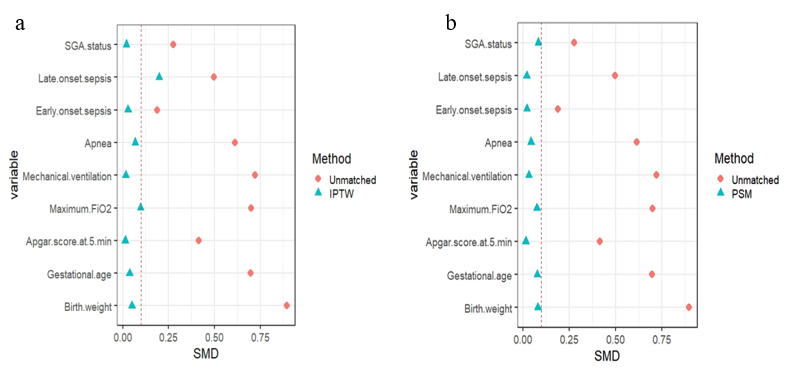

Supplement: Multimedia Appendix 6 [file pediatrics-v7-e60330-s006.png]

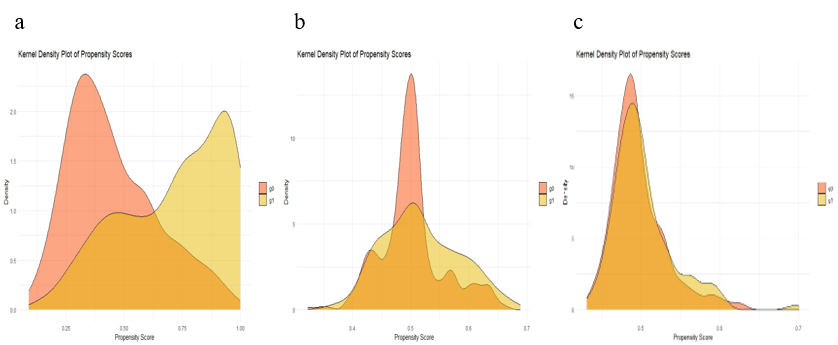

Supplement: Multimedia Appendix 7 [file pediatrics-v7-e60330-s007.png]
