# Supplementary material for: Red Blood Cell Transfusion for Incidence of Retinopathy of Prematurity: Prospective Multicenter Cohort Study
Source: JMIR Pediatr Parent. 2024 Sep 18;7:e60330. doi: 10.2196/60330 (PMC11425406; doi:10.2196/60330)
Supplement: Multimedia Appendix 8 [file pediatrics-v7-e60330-s008.docx]

Supplementary Table S6. The impact of RBC transfusion within 4 weeks on ROP incidence after IPTW.

|  | Nontransfusion group (N=833), n (%) | Transfusion group (N=838), n (%) | aOR^a^ (95% CI) | *P* value |
| --- | --- | --- | --- | --- |
| ROP | 214(25.7) | 316(37.8) | 1.92 (1.50, 2.27) | <.001 |
| ≥stage 2 ROP | 125(15.0) | 201(24.1) | 1.71 (1.27, 2.31) | <.001 |
| Severe ROP | 47(5.6) | 57(6.8) | 1.16 (0.69, 1.98) | .600 |
| ^a^aOR: adjusted odds ratio. Adjusted for gestational age, birth weight, 5-minute Apgar score, mechanical ventilation use, maximum oxygen concentration, early-onset sepsis, late-onset sepsis, apnea, and SGA. | | | | |
