# Supplementary material for: Red Blood Cell Transfusion for Incidence of Retinopathy of Prematurity: Prospective Multicenter Cohort Study
Source: JMIR Pediatr Parent. 2024 Sep 18;7:e60330. doi: 10.2196/60330 (PMC11425406; doi:10.2196/60330)
Supplement: Multimedia Appendix 9 [file pediatrics-v7-e60330-s009.docx]

Supplementary Table S7. The impact of RBC transfusion within 4 weeks on ROP incidence after PSM.

|  | Nontransfusion group (N=238), n (%) | Transfusion group (N=238) , n (%) | aOR^a^ (95% CI) | *P* value |
| --- | --- | --- | --- | --- |
| ROP | 48 (20.2) | 72 (30.3) | 1.81 (1.13, 2.92) | .015 |
| ≥stage 2 ROP | 26 (11.0) | 44 (18.5) | 2.08 (1.16, 3.81) | .016 |
| Severe ROP | 3 (1.3) | 7 (3.0) | 1.78 (0.36, 10.9) | .500 |
| ^a^aOR: adjusted odds ratio. Adjusted for gestational age, birth weight, 5-minute Apgar score, mechanical ventilation use, maximum oxygen concentration, early-onset sepsis, late-onset sepsis, apnea, and SGA. | | | | |
